# Detection and genotypic characterization of *Toxoplasma gondii* DNA within the milk of Mongolian livestock

**DOI:** 10.1007/s00436-019-06306-w

**Published:** 2019-04-13

**Authors:** E. Iacobucci, N. S. Taus, M. W. Ueti, L. Sukhbaatar, Z. Bastsukh, S. Papageorgiou, H. Fritz

**Affiliations:** 1 College of Veterinary Medicine, Washington State University, Pullman, WA 99164 USA; 2 Animal Disease Research Unit, Agricultural Research Service, U.S. Department of Agriculture, Pullman, WA 99164 USA; 3 Laboratory of Helminthology, Institute of Veterinary Medicine UlaanBaatar, UlaanBaatar, Mongolia; 4 School of Veterinary Medicine, University of California Davis, 1 Shields Avenue, Davis, CA 95616 USA

**Keywords:** *Toxoplasma gondii*, Bactrian camel, Mongolia, Milk, PCR

## Abstract

*Toxoplasma gondii* is a global, zoonotic parasite capable of infecting any warm-blooded host. Toxoplasmosis can cause a variety of illnesses including abortions and congenital defects in humans, sheep, and goats. Congenital toxoplasmosis is considered to have the highest global disease burden of any foodborne illness in humans. This study examined the potential role of milk as a route of *T. gondii* transmission between livestock and humans within Mongolian herders, a little-studied population which relies heavily on animals. Milk of Mongolian sheep, goats and Bactrian camels was tested for the presence of *T. gondii* DNA, and a survey was conducted to ascertain what behavioral and environmental factors were present that might potentiate *T. gondii* infection within these Mongolian communities. *T. gondii* DNA was detected in samples from one sheep and five camels. Sequence analysis of DNA from camel milk revealed that two were from potentially virulent *T. gondii* genotypes. This has implications for public health in the region, as milk is an extremely important source of nutrition and our survey results imply that some people believe consumption of raw camel milk carries health benefits. This is the first report of *T. gondii* DNA in Bactrian camel milk as well as the first genotypic characterization of *T. gondii* within Mongolia.

**Electronic supplementary material:**

The online version of this article (10.1007/s00436-019-06306-w) contains supplementary material, which is available to authorized users.

## Introduction

*Toxoplasma gondii* is a protozoan parasite capable of infecting any warm-blooded host. An estimated one third of the world human population is chronically infected (Weiss and Dubey 2009). *T. gondii*’s complex life cycle, environmentally resistant oocyst stage, and multiple routes of infection make it a successful and ubiquitous zoonotic pathogen that infects humans, companion and livestock animals, and wildlife species around the world. Few data are available concerning *T. gondii* in Asia, and almost none are available in Mongolia. The goal of this study was to determine if *T. gondii* DNA is present in the milk of Mongolian livestock and ascertain what behavioral and environmental factors are present that may potentiate *T. gondii* infection within this community.

There is conflicting information regarding the risk of human infection with *T. gondii* via consumption of unpasteurized milk. However, in goat milk, *T. gondii* has been shown to be viable and in high enough numbers to infect mice and cats, both in raw milk and in cold enzyme–produced cheese products (Dubey et al. 2014). Raw dromedary camel milk has been proven to contain organisms capable of infecting camel calves and mice (Boughattas 2017). Bactrian camels have been found positive for *T. gondii* by serological tests, but there is no published data currently about detection of *T. gondii* in their milk (Wang et al. 2013). For Mongolians practicing nomadic pastoralism, toxoplasmosis may currently be an unidentified health threat. The United Nation’s Food and Agricultural Organization reports that annual milk consumption per person in rural Mongolia was 200 kg liquid milk equivalents (LME) compared with only 50 kg LME in Mongolian urban centers in 2005 (with > 150 kg LME considered “high” by global comparison) (Setsgee Ser-Od and Dugdill 2010). Liquid milk and traditional milk products are extremely culturally important and considered sacred in some religious and cultural groups. Despite the population’s dependence on livestock and the intimate relationship between the health of herding families and that of their animals, limited data are published about zoonotic disease in Mongolia.

There are three primary routes of infection, including ingestion of sporulated oocysts in contaminated water or food, ingestion of tissue cysts from undercooked animal products (bradyzoites) and transplacental transmission (tachyzoites) (Tenter et al. 2000, Rico-Torres et al. 2016). *Toxoplasma* undergoes sexual reproduction only within its definitive felid hosts resulting in production of the environmentally stable oocyst (Dubey et al. 1970; Frenkel et al. 1970). When sporulated oocysts are ingested by a susceptible intermediate host, *T. gondii* is capable of infecting any nucleated cell type (Dubey et al. 1998; Ferguson and Hutchison 1987; Guimaraes et al. 2008; Sims et al. 1989). As a result of the formation of latent tissue cyst stages following infection, the host remains infected for life (Tenter et al. 2000). *T. gondii* virulence among the three clonal lineages has been extensively studied by experimental mouse infections, with type I isolates being highly virulent in mice and types II and III less virulent or avirulent, respectively. Although the majority of hosts infected with *T. gondii* never display overt clinical signs, the infecting genotype may be an important determinant of virulence and clinical outcome of infection. Furthermore, *T. gondii* infection may pose a major risk to the developing fetus when infected transplacentally and for immunocompromised individuals, irrespective of the infecting genotype. While the importance and extent of *T. gondii* transmission via milk is not currently known, the demonstration herein of *T. gondii* DNA in milk and its genotypic characterization are important preliminary steps in directing future research and developing food safety recommendations to herding families.

## Materials and methods

**Fig. 1:**
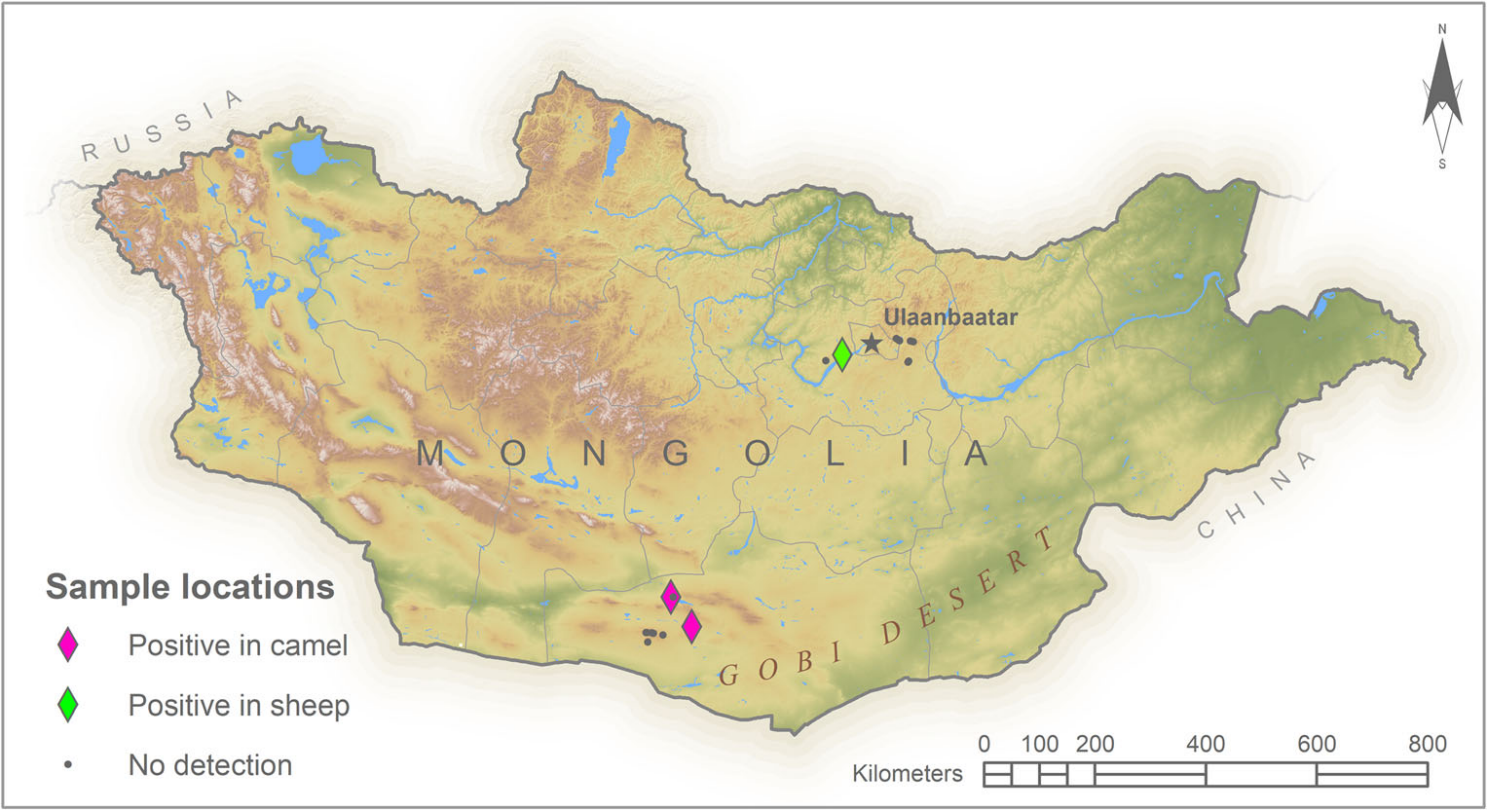
Study sites. Milk samples were collected at sites indicated by black dots. Pink triangles indicate the locations where *Toxoplasma* was detected in camel milk. The green triangle indicates the location where *Toxoplasma* was detected in sheep milk

Milk samples were collected from 19 herds within two Mongolian provinces, as shown in Fig. 1. Seven sampling sites were in Tuv province and nine in Omnigovi province. No more than 6 healthy animals per species were selected from each herd. A total of 59 Mongolian fuzzy goats, 58 Mongolian fat-tailed sheep, and 9 Bactrian camels were sampled.

DNA was extracted from 2 ml of milk from each individual animal using a DNeasy Blood & Tissue Kit (Qiagen) with some modifications to the manufacturer’s protocol. Each DNA product was first screened for DNA of the species by which the milk was produced as an internal control to ensure the extraction worked. Primers targeting the conserved gene DQA2 were used to screen for sheep and goat DNA while primers targeting the mitochondrial *cytb* gene were used to screen for Bactrian camel DNA. Samples which amplified internal control host species DNA were then screened in triplicate by PCR for *T. gondii* DNA targeting the ITS-1 and B1 genes by nested PCR. All primers are listed in online resource EMS_1. Positive amplicons were cleaned with ExoSAP-IT (ThermoFisher Scientific), sequenced to confirm amplicon specificity (Eurofins MWG Operon, Louisville, KY), and results were compared to published *T. gondii* genomic sequences in BLAST (blast.ncbi.nlm.nih.gov/Blast.cgi).

A survey regarding herd health, animal product use, and environmental factors was administered to the family head or heads in 16 of the 19 families visited. The survey questions and consent form are online resources EMS_ 2 and EMS_3.

## Results and discussion

**Fig. 2:**
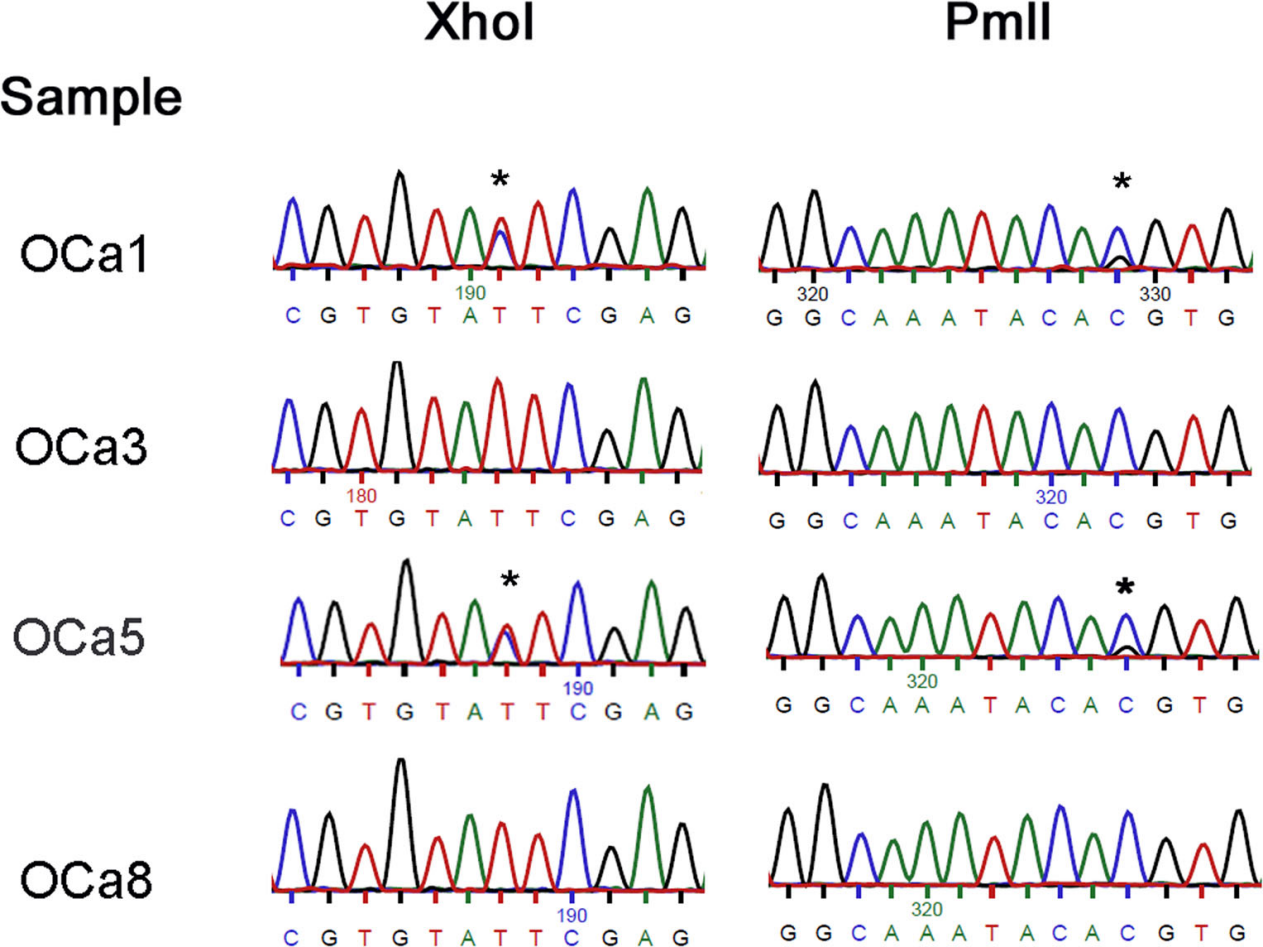
Chromatogram excerpts surrounding XhoI and PmlI sites within the B1 sequences of *Toxoplasma gondii* DNA extracted from camel milk. The codes on the left represent the individual animal and family. Asterisks are located over the nucleotides of interest which exhibit polymorphisms used to distinguish lineages. In OCa1 and OCa5, there are two nucleotide peaks at both locations. In OCa3 and OCa8, there is a single, strong nucleotide peak at these locations

*Toxoplasma gondii* DNA was detected in the milk of one sheep in Tuv province and eight Bactrian camels in Omnigovi. Genotyping of the *T. gondii* detected in four of the camels was done by examining bases numbered 366 and 504 in the published B1 sequence (GenBank accession number AF179871) (Grigg and Boothroyd 2001). At these locations, sequences from two camel samples had double peaks in the chromatogram, indicative of Toxoplasma type II and III lineages (Fig. 2). Chromatograms of *T. gondii* DNA from two other camel samples show only one nucleotide peak, which is indicative of a type I or atypical lineage (Fig. 2). Although the sample size is small, the results offer a glimpse of an unexpected ecology of *T. gondii* in the region. The detection of *T. gondii* in five of eight camels indicates that camels may play an important role in the *T. gondii* life cycle in the deserts of Asia. There is only one published report of a *T. gondii* seropositive Bactrian camel. It was located in Qinghai Province, China, approximately 1000 air km from this investigation’s milk positive camels (Wang et al. 2013). To the authors’ knowledge, this is the first report of *T. gondii* DNA detection within milk of Bactrian camels and the first genotypic characterization of *Toxoplasma gondii* within Mongolia. The detection of potential type I *T. gondii* DNA in camel milk is concerning given that it is considered more virulent than types II and III, as reflected by a lower LD_50_ in experimentally infected mice and their increased association with human congenital toxoplasmosis compared with the other lineages (Sibley and Boothroyd 1992).

Four surveys were completed in Terelj, three were completed in Hustai, and nine were completed in Gobi. More than half of the herders interviewed in this study believed domestic cats lived in near vicinity to their current homesteads, rising to over 80% when both domestic and wild cats (Siberian lynx, Pallas cat, and snow leopard) were included. Of the 16 herders interviewed, 56.25% reported a history of goat abortion in their herd, 18.75% reported a history of sheep abortions and one family (6.25%) reported a history of camel abortion. One serologic study examining *T. gondii* infection among sheep in seven northern provinces in Mongolia found infection in 16.57% by latex agglutination test and 24% by ELISA (Tumurjav et al. 2010). The prevalence of infection was low but ubiquitous in that study. This study’s survey reveals the elements to the *T. gondii* life cycle are present and the abortions reported could be part of pathologic effects on herd health. In addition to this study’s discovery of a potentially virulent genotype in Bactrian camel milk, one family reported consuming milk raw. The rest explained that the milk was always heated first, usually by simmering or boiling for a minimum of 15–20 min. People were generally aware that milk should be treated before consumption; however, the family reporting raw camel milk use explained their belief that it has health benefits which heated milk did not possess. The raw milk consumption reported in the survey is one possible route of transmission from livestock to humans.

Little data are available on the impact of zoonotic disease on the humans and livestock of Mongolia, including the impact of toxoplasmosis on humans in this country. For Mongolian herders, the risk factors exist for transmission, and according to our results, a potentially dangerous lineage of *T. gondii* is endemic in the area. Genotypic characterization of additional DNA samples would help to determine the extent to which virulent type I genotypes are circulating in the region. A better understanding of *T. gondii* biology within Bactrian camels would be extremely interesting, as this is an economically important species in several countries. Herders will benefit from knowledge of potential health threats and could direct efforts to build resilience mechanisms for their communities.

## Electronic supplementary material

ESM 1 Table of primers used in the study (DOCX 13 kb)

ESM 2 Survey questions asked in the study (PDF 127 kb)

ESM 3 **Consent form for study** Detection and genotypic characterization of *Toxoplasma gondii* DNA within the milk of Mongolian livestock (PDF 175 kb)

## References

[CR1] Boughattas S (2017). Toxoplasma infection and milk consumption: meta-analysis of assumptions and evidences. Crit Rev Food Sci Nutr.

[CR2] Dubey JP, Miller NL, Frenkel JK (1970). The *Toxoplasma gondii* oocyst from cat feces. J Exp Med.

[CR3] Dubey JP, Lindsay DS, Speer CA (1998). Structures of *Toxoplasma gondii* tachyzoites, bradyzoites, and sporozoites and biology and development of tissue cysts. Clin Microbiol Rev.

[CR4] Dubey JP (2014). Detection and survival of *Toxoplasma gondii* in milk and cheese from experimentally infected goats. J Food Prot.

[CR5] Ferguson DJ, Hutchison WM (1987). The host-parasite relationship of *Toxoplasma gondii* in the brains of chronically infected mice. Virchows Arch A Pathol Anat Histopathol.

[CR6] Frenkel JK, Dubey JP, Miller NL (1970). *Toxoplasma gondii* in cats: fecal stages identified as coccidian oocysts. Science.

[CR7] Grigg ME, Boothroyd JC (2001). Rapid identification of virulent type I strains of the protozoan pathogen *Toxoplasma gondii* by PCR-restriction fragment length polymorphism analysis at the B1 gene. J Clin Microbiol.

[CR8] Guimaraes EV, de Carvalho L, Barbosa HS (2008). Primary culture of skeletal muscle cells as a model for studies of *Toxoplasma gondii* cystogenesis. J Parasitol.

[CR9] Rico-Torres CP, Vargas-Villavicencio JA, Correa D (2016). Is *Toxoplasma gondii* type related to clinical outcome in human congenital infection? Systematic and critical review. Eur J Clin Microbiol Infect Dis.

[CR10] Setsgee Ser-Od T, Dugdill B (2010) Mongolia: rebuilding the dairy industry. In: Morgan N (ed) Smallholder dairy development: lessons learned in Asia. The Food and Agricultural Organization of the United Nations

[CR11] Sibley LD, Boothroyd JC (1992). Virulent strains of *Toxoplasma gondii* comprise a single clonal lineage. Nature.

[CR12] Sims TA, Hay J, Talbot IC (1989). An electron microscope and immunohistochemical study of the intracellular location of *Toxoplasma* tissue cysts within the brains of mice with congenital toxoplasmosis. Br J Exp Pathol.

[CR13] Tenter AM, Heckeroth AR, Weiss LM (2000). *Toxoplasma gondii*: from animals to humans. Int J Parasitol.

[CR14] Tumurjav B (2010). Serodiagnosis of ovine toxoplasmosis in Mongolia by an enzyme-linked immunosorbent assay with recombinant toxoplasma gondii matrix antigen 1. Jpn J Vet Res.

[CR15] Wang M, Wang YH, Meng P, Ye Q, Zhang DL (2013). *Toxoplasma gondii* infection in Bactrian camel (Camelus bactrianus) in China. Vet Parasitol.

[CR16] Weiss LM, Dubey JP (2009). Toxoplasmosis: a history of clinical observations. Int J Parasitol.

