# Supplementary material for: Detection and genotypic characterization of Toxoplasma gondii DNA within the milk of Mongolian livestock
Source: Parasitol Res. 2019 Apr 13;118(6):2005–8. doi: 10.1007/s00436-019-06306-w (PMC6521982; doi:10.1007/s00436-019-06306-w)
Supplement: Supplementary file 1 — Table of primers used in the study (DOCX 13 kb) [file 436_2019_6306_MOESM1_ESM.docx]

EMS_1 PCR primers used in study

|  | Gene | Reference |
| --- | --- | --- |
| Primer sequence |  |  |
| 5’ CTTCCTGCTCCTCACCCTCAC 3’ | *DQA2* | Zhou H., J Animal Science 2005 |
| 5’ AAAGAGAAGTAGAATGGTGGACACTT 3’ |  |  |
| 5’ TGCCATTTATTATCACAGC 3’ | *cytb* | Ji R., Anim Genet 2009 |
| 5’ TCTTCCCTGAGTCTTAGG 3’ |  |  |
| Outer ITS1DF, 5’ TACCGATTGAGTGTTCCGGTG 3’ | *ITS-1* | Rejmanek D., et. al, J Parasit 2010 |
| Outer ITS1DR 5’ GCAATTCACATTGCGTTTCGC 3’ |  |  |
| Inner ITS1diF 5’ CGTAACAAGGTTTCCGTAGG 3’ |  |  |
| Inner ITS1diR 5’ TTCATCGTTGCGCGAGCCAAG 3’ |  |  |
| Outer Pml/S1 5′ TGTTCTGTCCTATCGCAACG 3’ | *B1* | Grigg ME, Boothroyd JC, J Clin Micro 2001 |
| Outer Pml/AS1 5′ ACGGATGCAGTTCCTTTCTG 3’ |  |  |
| Inner Pml/S2 5′ TCTTCCCAGACGTGGATTTC 3’ |  |  |
| Inner Pml/AS2 5′ CTCGACAATACGCTGCTTGA 3’ |  |  |
