# Supplementary material for: Detection and genotypic characterization of Toxoplasma gondii DNA within the milk of Mongolian livestock
Source: Parasitol Res. 2019 Apr 13;118(6):2005–8. doi: 10.1007/s00436-019-06306-w (PMC6521982; doi:10.1007/s00436-019-06306-w)
Supplement: Supplementary file 2 — Survey questions asked in the study (PDF 127 kb) [file 436_2019_6306_MOESM2_ESM.pdf]

Date	Time

Region	
GPS Coordinates	
Family Code	

Which animals are in your herd?	Goats<br><br>Camels	Sheep<br><br>Horses	Cattle<br><br>Yaks
Is there a history of abortion in your herd?	Yes		No
○ If so, which animals?			
○ If so, at what stage of pregnancy?	Early Term	Mid Term	Late Term
Do you sell milk products?	Yes		No
○ If so, please explain			
Do you consume any milk raw or heat treat?	Heat Treat	Raw	Both
How do you treat your milk?			
Do you keep any domestic cats?	Yes		No
Do any domestic or wild cats live in the area?	Yes		No
○ If so, what type of wild cat?			
