# Supplementary material for: Detection and genotypic characterization of Toxoplasma gondii DNA within the milk of Mongolian livestock
Source: Parasitol Res. 2019 Apr 13;118(6):2005–8. doi: 10.1007/s00436-019-06306-w (PMC6521982; doi:10.1007/s00436-019-06306-w)
Supplement: Supplementary file 3 — Consent form for study Detection and genotypic characterization of Toxoplasma gondii DNA within the milk of Mongolian livestock (PDF 175 kb) [file 436_2019_6306_MOESM3_ESM.pdf]

Appenidx:

Livestock & Guardian Dog Research project

Herder Consent Form

We greatly appreciate your participation in this research project. This document constitutes the consent form that you give your permission for your animals to be enrolled as a study participant. All client and patient information is confidential.

Client Consent Form

Livestock and Guardian Dog Project

I, the undersigned, am the owner or authorized agent for the owner, and agree to enter my animal(s) in the study for livestock and guardian dogs. The study design, objectives and procedures of the study have been clearly explained to me, including blood, stool and milk collection. I understand that the risks are minimal and the same as if my animal was having an annual routine 'wellness' blood and physical examination.

I understand that the testing will be performed at no cost to the client or veterinarian. I give my permission to publish data generated from this study for the benefit of the scientific community. I understand that my animals, and I, the owner, will not be identified individually.

Following the discussion of the study protocol and after reading the above material, I voluntarily consent to enter my animals into this research project. I agree to participate in this study and will follow the instructions of the veterinarians as it applies to the protocol.

____Date:__________________

Signature of owner/agent

Email Phone

____ Date: __________________

Witness

I would like to receive the results for my animal(s) from the tests performed.

I would like to receive a summary of study results.
